# Editorial: Reproducibility and Rigour in Computational Neuroscience

**DOI:** 10.3389/fninf.2020.00023

**Published:** 2020-05-27

**Authors:** Sharon M. Crook, Andrew P. Davison, Robert A. McDougal, Hans Ekkehard Plesser

**Affiliations:** ^1^School of Mathematical and Statistical Sciences, School of Life Sciences, Arizona State University, Tempe, AZ, United States; ^2^Department of Integrative and Computational Neuroscience, Paris-Saclay Institute of Neuroscience, CNRS/Université Paris-Saclay, Gif-sur-Yvette, France; ^3^Department of Biostatistics and Center for Medical Informatics, Yale University, New Haven, CT, United States; ^4^Faculty of Science and Technology, Norwegian University of Life Sciences, Ås, Norway; ^5^Institute of Neuroscience and Medicine (INM-6), Jülich Research Centre, Jülich, Germany

**Keywords:** reproducible research, model sharing, model validation, replicability, code generation, model parameterization

## 1. Introduction

Independent verification of results is critical to scientific inquiry, where progress requires that we determine whether conclusions were obtained using a rigorous process. We also must know whether results are robust to small changes in conditions. Modern computational approaches present unique challenges and opportunities for these requirements. As models and data analysis routines become more complex, verification that is completely independent of the original implementation may not be pragmatic, since re-implementation often requires significant resources and time. Model complexity also increases the difficulty in sharing all details of the model, hindering transparency.

Discussions that aim to clarify issues around reproducibility often become confusing due to the conflicting usage of terminology across different fields. In this Topic, Plesser provides an overview of the usage of these terms. In previous work, Plesser and colleagues proposed specific definitions for repeatability, replicability, and reproducibility (Crook et al., 2013) that are similar to those adopted by the Association for Computing Machinery (2020). Here, Plesser advocates for the lexicon proposed by Goodman et al. (2016), which separates *methods reproducibility, results reproducibility*, and *inferential reproducibility*—making the focus explicit and avoiding the ambiguity caused by the similar meanings of the words reproducibility, replicability, and repeatability in everyday language. In the articles associated with this Topic, many authors use the terminology introduced by Crook et al. (2013); however, in some cases, opposite meanings for reproducibility and replicability are employed, although all authors carefully define what they mean by these terms.

## 2. Topic Overview

Although true independent verification of computational results should be the goal when possible, resources and tools that aim to promote the replication of results using the original code are extremely valuable to the community. Platforms such as open source code sharing sites and model databases (Birgiolas et al., 2015; McDougal et al., 2017; Gleeson et al., 2019) provide the means for increasing the impact of models and other computational approaches through re-use and allow for further development and improvement. Simulator-independent model descriptions provide a further step toward reproducibility and transparency (Gleeson et al., 2010; Cope et al., 2017; NineML Committee, 2020). Despite this progress, best practices for verification of computational neuroscience research are not well-established. Benureau and Rougier describe characteristics that are critical for all scientific computer programs, placing constraints on code that are often overlooked in practice. Mulugeta et al. provide a more focused view, arguing for a strong need for best practices to establish credibility and impact when developing computational neuroscience models for use in biomedicine and clinical applications, particularly in the area of personalized medicine.

Increasing the impact of modeling across neuroscience areas also requires better descriptions of model assumptions, constraints, and validation. When model development is driven by theoretical or conceptual constraints, modelers must carefully describe the assumptions and the process for model development and validation in order to improve transparency and rigor. For data driven models, better reporting is needed regarding which data were used to constrain model development, the details of the data fitting process, and whether results are robust to small changes in conditions. In both cases, better approaches for parameter optimization and the exploration of the sensitivity of parameters are needed. Here we see several approaches toward more rigorous model validation against experimental data across scales, as well as multiple resources for better parameter optimization and sensitivity analysis.

Viswan et al. describe FindSim, a novel framework for integrating experimental datasets with large multiscale models to systematically constrain and validate models. At the network level, considerable challenges remain over what metrics should be used to quantify network behavior. Gutzen et al. propose much needed standardized statistical tests that can be used to characterize and validate network models at the population dynamics level. In a companion study, Trensch et al. provide rigorous workflows for the verification and validation of neuronal network modeling and simulation. Similar to previous studies, they reveal the importance of careful attention to computational methods.

Although there are many successful platforms that aid in the optimization of model parameters, Nowke et al. show that parameter fitting without sufficient constraints or exploration of solution space can lead to flawed conclusions that depend on a particular location in parameter space. They also provide a novel interactive tool for visualizing and steering parameters during model optimization. Jȩdrzejewski-Szmek et al. provide a versatile method for the optimization of model parameters that is robust in the presence of local fluctuations in the fitness function and in high-dimensional, discontinuous fitness landscapes. This approach is also applied to an investigation of the differences in channel properties between neuron subtypes. Uncertainty quantification and sensitivity analysis can provide rigorous procedures to quantify how model outputs depend on parameter uncertainty. Tennøe et al. provide the community with Uncertainpy, which is a Python toolbox for uncertainty quantification and sensitivity analysis, and also provide examples of its use with models simulated with both NEURON (Hines et al., 2020) and NEST (Gewaltig and Diesmann, 2007).

Approaches and resources for reproducibility advocated by Topic authors cross many spatial and temporal scales, from sub-cellular signaling networks (Viswan et al.) to whole-brain imaging techniques (Zhao et al.). We discover that the NEURON simulation platform has been extended to include reaction-diffusion modeling of extracellular dynamics, providing a pathway to export this class of models for future cross-simulator standardization (Newton et al.). We also see how reproducibility challenges extend to other cell types such as glia as well as subcortical structures (Manninen et al.). At the network level, Pauli et al. demonstrate the sensitivity of spiking neuron network models to implementation choices, the integration timestep, and parameters, providing guidelines to reduce these issues and increase scientific quality. For spiking neuron networks specifically geared toward machine learning and reinforcement learning tasks, Hazan et al. provide BindsNET, a Python package for rapidly building and simulating such networks for implementation on multiple CPU and GPU platforms, promoting reproducibility across platforms.

And finally, Blundell et al. focus on one approach to address the challenges for reproducibility that arise due to increasing model complexity, which relies on high-level descriptions of complex models. These high-level descriptions require translation to code for simulation and visualization, and the use of code generation to automatically translate description into efficient code enhances standardization. Here, authors summarize existing code generation pipelines associated with the most widely-used simulation platforms, simulator-independent multiscale model description languages, neuromorphic simulation platforms, and collaborative model development communities.

## 3. Outlook

In this Research Topic, researchers describe a wide range of challenges for reproducibility and rigor, as well as efforts to address them across areas of quantitative neuroscience. These include best practices that should be employed in implementing, validating, and sharing computational results; fully specified workflows for complex computational experiments; a range of tools supporting scientists in performing robust studies; and a carefully defined terminology. In view of the strong interest in the practices, workflows, and tools for computational neuroscience documented in this Research Topic, and their availability to the community, we are optimistic that the future of computational neuroscience will be increasingly rigorous and reproducible.

## Author Contributions

SC, AD, RM, and HP all contributed equally to determining and approved the content of this editorial. SC wrote the content of this editorial.

## Conflict of Interest

The authors declare that the research was conducted in the absence of any commercial or financial relationships that could be construed as a potential conflict of interest.
